# Using Chat and Text Technologies to Answer Sexual and Reproductive Health Questions: Planned Parenthood Pilot Study

**DOI:** 10.2196/jmir.2619

**Published:** 2013-09-20

**Authors:** Margaret M Giorgio, Leslie M Kantor, Deborah S Levine, Whitney Arons

**Affiliations:** ^1^Department of Nutrition, Public Health, and Food StudiesNew York UniversityNew York, NYUnited States; ^2^Planned Parenthood Federation of AmericaNew York, NYUnited States

**Keywords:** reproductive health, sexual health, public health, text messaging, instant messaging, abortion seekers, sexually transmitted diseases, pregnancy, emergency contraception, Internet

## Abstract

**Background:**

Teens and young adults in the United States are in need of sexual and reproductive health information, as evidenced by elevated rates of sexually transmitted infections (STIs), pregnancy, and births among this population. In-person sexuality education programs are helpful, but they are unlikely to rapidly accommodate teens and young adults in a moment of crisis. Evidence suggests that technologies such as instant messaging (IM) and text messaging may be effective ways to provide teens and young adults with sexual and reproductive health information. In September 2010, Planned Parenthood Federation of America launched a text and IM program designed to provide immediate answers to urgent sexual and reproductive health questions from a reliable and confidential source and to link young people to sexual and reproductive health services if needed.

**Objective:**

To assess whether this program is successful in reaching the target population, whether user characteristics vary by mode (IM vs text), and whether mode is associated with reaching individuals with high levels of worry or reducing worry postchat.

**Methods:**

Data were collected from prechat and postchat surveys for all IM and text message conversations between September 2010 and August 2011. A bivariate analysis was conducted using chi-square tests for differences in the main covariates by mode of conversation. In the multivariable analysis, logistic regression was used to identify factors that were independently associated with prechat levels of worry and changes in worry postchat.

**Results:**

A total of 32,589 conversations occurred during the program’s first year. The odds of feeling very worried prechat were highest for IM users (adjusted odds ratio [AOR] 1.43, 95% CI 1.20-1.72), users 17 years and younger (AOR 1.62, 95% CI 1.50-1.74), Latino/Hispanic users (AOR 1.36, 95% CI 1.27-1.46), and black users (AOR 1.40, 95% CI 1.30-1.50). After controlling for the study covariates, there was no significant difference in the odds of feeling better (less worried) postchat between IM and text message users. Feeling better postchat was associated with being younger (≤17 years: AOR 1.42, 95% CI 1.17-1.72; 18-24 years: AOR 1.20, 95% CI 1.02-1.42), being Latino/Hispanic (AOR 1.31, 95% CI 1.10-1.55), reporting that the service was very helpful (AOR 3.47, 95% CI 3.24-4.32), and asking about emergency contraception (AOR 1.35, 95% CI 1.13-1.61). The odds of feeling better were lowest for users with questions about STIs (AOR 0.61, 95% CI 0.47-0.78).

**Conclusions:**

The results from the process evaluation suggest that the program was able to provide informational support to vulnerable groups, such as teens and racial minorities, in moments of particular worry. Differences between the IM and text message users reveal that each mode appeals to a different population and that both are necessary to reach a diverse audience.

## Introduction

There are approximately 43 million people aged 15-24 years in the United States, and their need for sexual and reproductive health information is evidenced by the elevated rates of sexually transmitted infections (STIs), pregnancies, and births among this population [1]. Although teens and young adults represent only 25% of the sexually active population, those aged 15-24 years account for nearly half of all STI diagnoses each year [2]. The pregnancy rate among those aged 15-19 years in the United States continues to be one of the highest in the developed world—more than twice as high as rates in Canada and Sweden [3]. Approximately 82% of pregnancies among those aged 15-19 years are unintended, which accounts for approximately 20% of all unintended pregnancies in the United States annually [4]. However, this rate has declined from previous years. In 2008, the pregnancy rate among those aged 15-19 years in the United States was 67.8 per 1000, which is the lowest it has been in 30 years [5].

Adverse sexual and reproductive health outcomes are not uniformly distributed among racial and ethnic subgroups in the United States. Recent STI surveillance efforts by the Centers for Disease Control and Prevention (CDC) have shown that rates for chlamydia, gonorrhea, and syphilis among those aged 10-24 years are significantly higher for blacks compared to whites [6,7]. In 2010, chlamydia rates were 13 times higher and gonorrhea rates were 37 times higher among black males aged 15-19 years than among white males of the same age group [7]. For males aged between 20-24 years, chlamydia rates were 8 times higher and gonorrhea rates were 23 times higher among blacks than among whites [7]. Similar differences exist by racial and ethnic group. The 2006-2010 results of the National Survey of Family Growth revealed that among females aged 15-19 years, 21% of Latino/Hispanic women reported using no method of contraception during last intercourse as compared with 19% of blacks and 11% of whites [8]. Racial disparities in condom use were also found among males of the same age group, with 31% of sexually active Latino/Hispanic males reporting never using condoms in the past 4 weeks compared to 21% of white and 16% of black males [8]. Pregnancy rates are also higher among black and Latino/Hispanic teens and young adults. In 2008, the highest pregnancy rates among those aged 15-19 years were reported among blacks (117 births per 1000) and Latino/Hispanics (107 births per 1000) compared to a rate of 43 per 1000 for white youth in the same age range [5,9]. This trend remained consistent for women aged 20-24 years; rates per 1000 population were 259 among black women and 245 among Latino/Hispanic women, compared with 63 among whites [6]. In the United States, black and Latino/Hispanic women have also been shown to experience higher rates of unintended pregnancy and abortion than white women do, a trend that persists after controlling for levels of income [10].

The high rates of STIs and unintended pregnancies among teens and young adults in the United States point to an increased need to provide sexual and reproductive health information to this group. Sexuality education programs have traditionally been implemented in schools, health centers, and community settings, yet these venues and methods are lacking in 2 respects. First, not everyone gets sexuality education, and those that do may not receive accurate, effective, or timely sexuality education [11-14]. Second, although in-person sexuality education programs can provide information and support, it is doubtful that they can rapidly accommodate teens and young adults when they are in a moment of crisis. This is especially problematic for time-sensitive issues, such as the window in which emergency contraception is most effective after unprotected sex. In addition, the longer an individual remains in a state of crisis, the higher her levels of anxiety and worry can become. Anxiety has been shown to be a barrier to health-seeking behavior [15,16].

New approaches to sexuality education are needed that can meet the needs of those not receiving adequate sexuality education in schools and communities and may meet needs in a moment of high worry. Technology can play an important role. Two of these types of technologies are instant messaging (IM) and short message service (SMS) text messaging. In 2006, it was estimated that 69 million people in North America used IM [17]. Between 2000 and 2004, researchers estimated an annual growth rate of IM users of approximately 29% [18], with the highest growth occurring among individuals between the ages of 13 and 29 years [19,20]. Text messaging has also become a major communication tool for teens and young adults. Research conducted by the Pew Internet & American Life Project revealed that 95% of those aged 18-29 years and 72% of those aged 12-17 years use the text messaging feature on their phones [21,22]. Among these groups, girls aged 14-17 years are the heaviest users, averaging approximately 100 messages per day [21]. Three-quarters of youth aged 12-17 years own mobile phones. Almost 90% of mobile phone users aged 12-17 years regularly send SMS text messages. Research also shows that those aged 14-17 years typically send or receive approximately 60 SMS text messages per day [23].

Existing evidence suggests that the use of IM and SMS text messaging may be an effective way to provide teens and young adults with sexual and reproductive health information [21,22]. Embarrassment and concerns about confidentiality can act as barriers to teens seeking sexual and reproductive health information from parents, peers, doctors, and/or other adults [24-26]. Teens and young adults may prefer the anonymity that instant and SMS text messaging provide. A recent review of the literature found that SMS text messaging has been used to promote sexual and reproductive health in a variety of ways, including communication between health clinics and patients, partner notification and contact tracing, contraception reminders, and sexuality education [27]. The review noted that some evidence of the effectiveness of this method exists; however, very few of the described studies had been formally evaluated [27]. An internal study conducted for Planned Parenthood Federation of America in 2007 of youth and young adults aged 13-29 years revealed that 32% of the study sample reported that they were likely or very likely to use IM if it was made available on the Planned Parenthood Federation of America website. Further, an email feature on Planned Parenthood Federation of America’s teen-targeted website received more than 20,000 questions in 2008. This high volume suggested to Planned Parenthood Federation of America staff that teens desired anonymity and confidentiality, but also wanted to get personalized answers to their questions. The answers to most of these questions are directly available on the website. However, users’ emails indicated a desire to communicate about their unique situations and get what they perceived to be unique answers. Both IM and texting are both vehicles through which users can be anonymous and also get highly personalized responses.

In light of these considerations, Planned Parenthood Federation of America launched a texting and IM program in September 2010 targeted at teens and young adults aged between 15-24 years who have an urgent sexual or reproductive health need. The goals of this program are to (1) give immediate answers to urgent sexual and reproductive health questions from a reliable and confidential source, and (2) link young people to sexual and reproductive health services if needed. The program additionally aims to reach groups experiencing health disparities, particularly black and Latino/Hispanic teens and young adults.

The program is focused on the following topic areas: emergency contraception, pregnancy tests, abortion, and STI testing. These 4 were chosen for a number of reasons. Topics were chosen based on Web analytics on the Planned Parenthood website, which included frequency of searches of these topic areas on the website, searches utilizing search engines (eg, Google, Yahoo) that led visitors to the website, and volume of page views containing this content. Topic areas were also chosen based on an analysis of questions received via the Ask the Expert email feature on the Planned Parenthood website. Further, the language used in the emails indicated high levels of worry that could be an obstacle to seeking help. Providing immediate personal interaction might alleviate this worry and allow the user to take a positive health-seeking action. Lastly, these issues are often time-sensitive and IM and texting allow a direct connection to users right when the information is needed, thereby filling a gap that sexuality education programs and health centers with limited hours are unable to meet.

The program operates as a national sexual and reproductive health hotline that, as of December 2012, operates from 9 am to midnight (ET) Monday to Thursday, 9 am to 10 pm (ET) on Fridays, 9 am to 5 pm (ET) on Saturdays, and from 2 pm to midnight (ET) on Sundays. By clicking the IM function on the Planned Parenthood Federation of America website or by sending a SMS text message, program users interact with live, trained customer service agents. Agents use a bank of more than 900 scripted responses to provide health information, correct misconceptions, and provide contact information for a Planned Parenthood health center when warranted.

To create the bank of standardized responses, likely questions were identified through archives of questions submitted to Ask the Expert on the Planned Parenthood website, Columbia University’s Go Ask Alice email feature, Ask Dr Cullins on the Planned Parenthood website, and other frequently asked question (FAQ) documents and archives from Planned Parenthood affiliates. Responses to these questions were then gathered from Planned Parenthood pamphlets, websites, and training materials. Further responses were developed by a team of external health writers with expertise in the topic areas and edited by Planned Parenthood Federation of America staff. In the first months of the program, staff actively reviewed transcripts to identify gaps and improve the existing responses. The program maintains an ongoing system for transcript review and content improvement.

Agents are trained on the substantive topics, the use of scripted responses, and how to respond compassionately and appropriately. Agents provide medical information, but do not provide medical advice or diagnoses. They use scripted messages that contain simple counseling content, but are trained to remain on the script and refer users to health centers for more specific or in-depth counseling. Agent performance is monitored and evaluated by staff with advanced sexual health training to ensure high-quality responses that meet the goals of the program. In addition, agents work in a central location and agents that are more skilled are assigned to the task of monitoring conversations as they are happening and providing guidance as needed.

For the pilot phase of the program, Planned Parenthood Federation of America promoted the service on its traditional and mobile websites, which attract 3 million visitors monthly, as well as on 2 MTV television shows: *16 and Pregnant* and *Teen Mom*.

This paper describes the results from a process evaluation of the first year of the IM and texting program. The process evaluation had 4 main research questions:

1. Was the program successful in reaching the target population, teens and young adults (15-24 years) and black and Latino/Hispanic teens and young adults?
2. Did the program reach the target population when they had high levels of worry?
3. Did user characteristics (sociodemographic, question topic, and level of worry) vary by mode (IM vs texting)?
4. Was IM or texting more likely to reach individuals when they had high levels of worry and to reduce user-reported worry postconversation?

## Methods

### Data Sources

Data for this process evaluation were collected from September 2010 until August 2011. All SMS text message and IM conversations that occurred during this period were included in the analysis (N=32,589). Data for this analysis came from 3 main sources. The first was a short prechat survey that was offered to all users prior to being connected to an agent. Because of differences between the IM and SMS text service providers, completion of the prechat survey was required for IM users whereas it was offered, but not required, for individuals communicating via texting. The second data source was a postchat survey. Technological limitations of the software program prevented the postchat survey from being offered to all users or from requiring completion by those who were offered it. Lastly, program agents were instructed to fill out a postchat survey for each interaction.

Two service providers, 1 for IM and the other for SMS text messaging, were contracted by Planned Parenthood Federation of America to provide the technological platforms for the program. Each of these providers maintained databases containing data from the prechat, postchat, and agent surveys. Neither database contained a complete list of all variables and cases. Therefore, the 2 datasets were combined to create the final dataset used for this analysis. This dataset was then cleaned to remove duplicate variables and cases.

### Variables

Table 1 contains a list of the variables that were collected through each of the 3 data sources, broken down by conversation mode (IM vs texting). A number of demographic characteristics were assessed, including age, gender, race, and zip code. For age, gender, and race, the participant was offered a precategorized list and asked to choose the 1 category that best described them. For the purposes of this analysis, age categories were collapsed to reflect 3 groups: teens (≤17 years), young adults (18-24 years), and adults (≥25 years). Because of the small number of participants who identified as transgender (n=148), these individuals were not included in the final sample. Although race was assessed through the prechat survey for IM users, texting users were not asked for their race until the postchat survey. The race variable was collapsed into 4 categories: white, black, Latino/Hispanic, and other.

For IM users, question topic was assessed through the agent survey and the prechat survey. To simplify the user experience for texting users, they were not asked to describe their question topic. The respondent or agent was asked to choose from the following list: abortion, STI testing, pregnancy testing, emergency contraception, and other. To construct the question topic variable, the agent-reported question categories were used as the default. In most cases (23,138/32,589, 70.99%), the responses about the question topic area from the program user and agent were in agreement. An informal qualitative analysis of a sample of transcripts from conversations with discordant responses revealed that the agent responses were more reliable than user responses. In addition, the agent-reported topic was the only data available for texting users. In the case of missing data from the agent survey (3585/32,589, 11.00%), IM user responses were used instead.

To assess whether an individual was in a moment of crisis, users were asked to report how worried they felt about their question. Level of worry was assessed twice: first in the prechat survey and again in the postchat survey. Participants were asked to report if they felt very worried, somewhat worried, or not at all worried. A dichotomous variable was created to determine changes in worry level from prechat to postchat, and users were categorized as either less worried, no change, or increased worry. Less worried could mean that someone went from feeling very worried to somewhat worried, from very worried to not at all worried, or from somewhat worried to not at all worried. Similarly, no change could represent an individual who reported any of the 3 levels of worry prechat, as long as they reported the same level of worry postchat. The no change and increased worry response options were combined due to the small number of users (329/32,589, 5.83%) who reported an increased level of worry postchat.

Users were asked in the postchat survey to rate the helpfulness of the service, with response categories of very helpful, somewhat helpful, somewhat unhelpful, and not at all helpful. In the multivariable models, these categories were collapsed to compare individuals who found the program very helpful to all other users.

### Statistical Analysis and Multivariable Models

Ethical clearance for this analysis was obtained from the Allendale Investigational Review Board. To investigate the main research questions, descriptive statistics for the final sample were run for all variables. Next, a bivariate analysis was conducted using Pearson chi-square test for significance for differences in the main covariates by mode of conversation. In the multivariable analysis, logistic regression was used to identify factors that were independently associated with prechat levels of worry and changes in worry postchat. Models 1 and 3 present the unadjusted odds for the relationships between conversation type and the 2 worry measures. Models 2 and 4 present the adjusted odds ratios, controlling for the other study covariates.

**Table 1:** Questions, answer categories, associated variables, and differences by conversation for the study data sources.

Variable domain		Instant messaging	Texting
**Prechat survey**			
	Gender	Your gender?	Please text your age, sex, and zip code
		Women	(open ended)
		Man	
		Transgender	
	Age	Your age?	Please text your age, sex, and zip code
		11 and under	(open ended)
		12-14	
		15-17	
		18-24	
		25-30	
		31-50	
		51 or older	
	Zip code	Your zip code?	Please text your age, sex, and zip code
		(open ended)	(open ended)
	Race	What best describes your race/ethnicity?	(Collected in postchat survey)
		White	
		Black	
		Latino/Hispanic	
		American Indian/Alaska Native	
		Asian/Pacific Islander	
		Biracial/multiracial	
		Other	
	Question topic	You may have more than one question, but what is the main thing you want to chat about today?	(Collected in agent survey)
		Abortion	
		STD testing	
		Pregnancy tests	
		Morning-after pill (emergency contraception)	
		Other	
	Prechat worry level	How are you feeling right now?	Before we get started, please tell us how you are feeling right now
		Very worried	Very worried
		Somewhat worried	Somewhat worried
		Not at all worried	Not at all worried
**Postchat survey**			
	Postchat worry level	How are you feeling now?	To help us know how well we’re doing, please answer three questions. First, how are you feeling now?
		Very worried	Very worried
		Somewhat worried	Somewhat worried
		Not at all worried	Not at all worried
	Helpfulness	How helpful were we?	How helpful were we?
		Very helpful	Very helpful
		Somewhat helpful	Somewhat helpful
		Somewhat unhelpful	Somewhat unhelpful
		Not at all helpful	Not at all helpful
	Race	(Collected in prechat survey)	What best describes your race/ethnicity?
			American Indian
			Asian/Pac Islander
			Black
			Latino/Hispanic
			Multiracial
			White
			Other
**Agent survey (same for both IM and texting)**			
	Appointment offered	For chatter/texter in participating health center area, did you OFFER an appointment?	For chatter/texter in participating health center area, did you OFFER an appointment?
		Yes, no, N/A	Yes, no, N/A
	Appointment booked	For chatter/texter in participating health center area, did you BOOK an appointment?	For chatter/texter in participating health center area, did you BOOK an appointment?
		Yes, no, N/A	Yes, no, N/A
	Contact info offered	For chatter/texter NOT in participating health center area, did you OFFER local PP contact information?	For chatter/texter NOT in participating health center area, did you OFFER local PP contact information?
		Yes, no, N/A	Yes, no, N/A
	Contact info provided	For chatter/texter NOT in participating health center area, did you PROVIDE local PP contact information?	For chatter/texter NOT in participating health center area, did you PROVIDE local PP contact information?
		Yes, no, N/A	Yes, no, N/A
	Chatter ask for appointment	For chatter/texter NOT in participating health center area, did the chatter ask explicitly for an appointment?	For chatter/texter NOT in participating health center area, did the chatter ask explicitly for an appointment?
		Yes, no, N/A	Yes, no, N/A
	Question topic	What was discussed? Select all that apply.	What was discussed? Select all that apply.
		Abortion	Abortion
		Emergency contraception	Emergency contraception
		Pregnancy testing	Pregnancy testing
		STI testing	STI testing
		Other	Other

## Results

Descriptive statistics are displayed in Table 2. There was a total of 32,589 conversations that occurred during the first year of the program, but the n’s vary for each variable due to missing data. Users were most commonly white (46.17%, 12,119/26,250), aged 18-24 years (51.20%, 16,485/32,195), and female (89.29%, 28,575/32,002). Although not in the majority, the program reached substantial numbers of Latino/Hispanic and black individuals during the first year. Specifically, the next largest categories were Latino/Hispanic (18.59%, 4881/26,250) and black (16.87%, 4429/26,250) users. The other category accounted for 18.37% (4821/26,250) of the sample and comprised individuals who identified as American Indian/Alaska Native, Asian/Pacific Islander, biracial/multiracial, or other. Almost one-quarter of the users (23.30%, 7500/32,195) were aged 17 years or younger. Questions about abortion were the most prevalent (44.65%, 13,617/30,498). Pregnancy testing and emergency contraception were the next most common topics, both individually representing 17.33% and 17.35% (5284/30,498 and 5292/30,498, respectively) of conversations. The least common topic was STI testing (9.21%, 2810/30,498). Most program users reported at least some level of worry prechat, with 43.22% (13,365/30,921) reporting feeling very worried and 45.43% (13,953/30,921) reporting feeling somewhat worried. Information for a Planned Parenthood health center was provided in just over half (54.57%, 13,318/24,231) of conversations.

The overall response rate for the postchat survey was 17.67% (5759/32,589). Among the users who completed the survey, most found this service to be very helpful (61.91%, 3559/5749). Levels of worry were lower postchat. Only 19.33% (1113/5758) reported feeling very worried after interacting with an agent, as opposed to 51.93% (2990/5758) feeling somewhat worried and 28.74% (1655/5758) feeling not at all worried. The variable that was created to capture changes in level of worry revealed that 37.90% (2140/5647) of users reported feeling less worried immediately after the chat had concluded. Although slightly more than half (56.28%, 3178/5647) reported no change, a small group (5.83%, 329/5647) reported an increase in their level of worry postchat.

Using Pearson chi-square test for significance, statistical differences between texting and IM users were found for most of the major covariates (Table 2). Texting users were more likely than IM users to be male (14.15% vs 10.19%, *P*<.001), Latino/Hispanic (24.65% vs 18.41%, *P*<.001), and aged 17 years or younger (39.02% vs 20.90%, *P*<.001) than individuals using IM. There were also significant differences by question topic areas (*P*<.001). Although 46.61% (13,020/27,933) of IM users asked questions about abortion, it accounted for only 23.27% (597/2565) of questions from texting users. On the other hand, texting users were more likely than IM users to ask about STI testing (16.49% vs 8.55%, *P*<.001) or had questions categorized as other (22.38% vs 10.46%, *P*<.001). Texting users were more likely than IM users to report that the service was very helpful (73.01% vs 60.13%, *P*<.001). No statistically significant differences were found between texting and IM users in the frequency that Planned Parenthood health center information was provided.

Overall, there was a statistically significant difference in which texting users appeared to be slightly less worried than IM users (Table 2). In the prechat survey, IM users were more likely than texting users to report being very worried (43.70% vs 38.79%, *P*<.001), whereas texting users more commonly reported feeling not at all worried compared to IM users (15.52% vs 11.24%, *P*<.001). There is a similar pattern in the postchat survey. Although a comparable proportion of IM and texting users reported feeling very worried, a larger percentage of IM users reported being somewhat worried (53.03% vs 45.14%, *P*<.001). Feeling not at all worried was more common among texting versus IM users (35.16% vs 27.70%, *P*<.001) in the postchat survey. Looking at differences between texting and IM users in the changes in level of worry variable, a larger percentage of texting users reported a higher level of worry postchat (8.25% vs 5.49%, *P*<.001). There was no difference in the percentage of users who reported feeling less worried.

As shown in Table 3, Pearson chi-square test for significance was also used to reveal significant differences in user-reported program helpfulness postchat by question topic (*P*<.001). Among users with questions concerning STIs, only 53.31% (314/589) reported that the program was very helpful. By comparison, 70.48% of users (795/1128) with questions about emergency contraception reported that the chat was very helpful.

Table 4 provides the unadjusted and multivariable estimates of the odds ratios (OR) for the relationship between conversation mode and 2 different measures of worry. Models 1 and 2 use prechat worry (feeling very worried before interacting with a program agent as compared to feeling somewhat worried or not at all worried) as the outcome variable. The unadjusted odds ratio (UOR) reveals that IM users were more likely to report feeling very worried than texting users prechat (UOR 1.22, 95% CI 1.13-1.32). After controlling for the other study covariates, the adjusted odds ratio (AOR) for this relationship increased to 1.43 (95% CI 1.20-1.72). In Model 2, all the coefficients were significant at a *P*<.01 with the exception of gender. The odds of feeling very worried were highest for users aged 17 years and younger (AOR 1.62, 95% CI 1.50-1.74), Latino/Hispanic users (AOR 1.36, 95% CI 1.27-1.46), and black users (AOR 1.40, 95% CI 1.30-1.50). Users with questions about abortion were the most worried, with the odds of feeling very worried prechat being 65% less for users with questions about STI testing, 60% less for users whose questions fell into the other category, 43% less for questions about emergency contraception, and 26% less for questions about pregnancy testing.

Models 3 and 4 use the change in worry variable as the outcome, and the coefficients estimate the odds of feeling better (ie, less worried) postchat compared to reporting the same or increased level of worry. These models only pertain to the 17.67% of the total sample that completed the postchat survey (5759/32,589). The UOR for the relationship between conversation type and postchat worry revealed no significant difference between IM and texting users in the likelihood of feeling better postchat (UOR 0.99, 95% CI 0.84-1.16). After controlling for the other study covariates, this finding remained nonsignificant. However, there were significant relationships between other study covariates and changes in worry level postchat. Compared to the oldest users, individuals in the younger age groups were more likely to report lower levels of worry postchat (≤17 years: AOR 1.42, 95% CI 1.17-1.72; 18-24 years: AOR 1.20, 95% CI 1.02-1.42). Feeling better postchat was associated with users reporting being Latino/Hispanic (AOR 1.31, 95% CI 1.10-1.55) and reporting that the service was very helpful (AOR 3.47, 95% CI 3.24-4.32). Using abortion as a reference, users with questions about emergency contraception were most likely to report feeling less worried postchat (AOR 1.35, 95% CI 1.13-1.61). Conversely, the odds of feeling better were lowest for users with questions about STIs compared to abortion questions (AOR 0.61, 95% CI 0.47-0.78).

**Table 2:** Characteristics of program users and conversations by mode of conversation, instant messaging (IM), and texting.

Variables		All users (N=32,589)		IM (n=27,939)		Texting (n=4650)		*P* value
**Question topic** ^a^								
	Abortion	13,617	44.65	13,020	46.61	597	23.27	<.001
	Pregnancy testing	5284	17.33	4761	17.04	523	20.39	
	Emergency contraception	5292	17.35	4844	17.34	448	17.47	
	STI testing	2810	9.21	2387	8.55	423	16.49	
	Other	3495	11.46	2921	10.46	574	22.38	
**Gender** ^b^								
	Female	28,575	89.29	24,959	89.81	3616	85.85	<.001
	Male	3427	10.71	2831	10.19	596	14.15	
**Race** ^c^								
	White	12,119	46.17	11,789	46.30	330	41.93	<.001
	Black	4429	16.87	4320	16.97	109	13.85	
	Latino/Hispanic	4881	18.59	4687	18.41	194	24.65	
	Other	4821	18.37	4667	18.33	154	19.57	
**Age (years)** ^d^								
	≤17	7500	23.30	5839	20.90	1661	39.02	<.001
	18-24	16,485	51.20	14,361	51.40	2124	49.89	
	≥25	8210	25.50	7738	27.70	472	11.09	
**Prechat level of worry** ^e^								
	Very worried	13,365	43.22	12,208	43.70	1157	38.79	<.001
	Somewhat worried	13,953	45.12	12,590	45.06	1363	45.69	
	Not at all worried	3603	11.65	3140	11.24	463	15.52	
**Health center information given** ^f^								
	Yes	13,224	54.57	11,993	54.48	1231	55.50	.40
	No	11,007	45.43	10,020	45.52	987	44.50	
**Postchat questions** ^g^								
	Response rate	5759	17.67	4956	17.74	802	17.25	.42
**Postchat level of worry** ^h^								
	Very worried	1113	19.33	955	19.27	158	19.70	<.001
	Somewhat worried	2990	51.93	2628	53.03	362	45.14	
	Not at all worried	1655	28.74	1373	27.70	282	35.16	
**Changes in level of worry** ^i^								
	Less worried	2140	37.90	1876	37.85	264	38.21	.01
	No change	3178	56.28	2808	56.66	370	53.55	
	More worried	329	5.83	272	5.49	57	8.25	
**Helpfulness** ^j^								
	Very helpful	3559	61.91	2980	60.13	579	73.01	<.001
	Less than very helpful	2190	38.09	1976	39.23	214	26.99	

^a^All users: n=30,498; IM: n=27,933; texting: n=2565.

^b^All users: n=32,002; IM: n=27,790; texting: n=4212.

^c^All users: n=26,250; IM: n=25,463; texting: n=787.

^d^All users: n=32,195; IM: n=27,938; texting: n=4257.

^e^All users: n=30,921; IM: n=27,938; texting: n=2983.

^f^All users: n=24,231; IM: n=22,013, texting: n=2218.

^g^All users: n=32,589; IM: n=27,939; texting: n=4650.

^h^All users: n=5758; IM: n=4956; texting: n=802.

^i^All users: n=5647; IM: n=4956; texting: n=691.

^j^All users: n=5749; IM: n=4956; texting: n=793.

**Table 3:** Differences in user-reported program helpfulness postchat by question topic (n=5626).

Helpfulness	Question topic area										
	Abortion (n=2168)		Pregnancy testing (n=1175)		Emergency contraception (n=1128)		STI testing (n=589)		Other (n=566)		*P* value
	n	%	n	%	n	%	n	%	n	%	
Very helpful	1339	61.76	716	60.94	795	70.48	314	53.31	312	55.12	<.001
Less than very helpful	829	38.24	459	39.06	333	29.52	275	46.69	254	44.88	

**Table 4:** Unadjusted (UOR) and adjusted (AOR) logistic regression estimates of the relationship between conversation mode and worry measures.

Variables		Odds of reporting							
		Very worried prechat^a^ (n=25,882)				Reduced worry postchat^b^ (n=4359)			
		UOR	95% CI	AOR	95% CI	UOR	95% CI	AOR	95% CI
**Conversation mode**									
	Texting	1.00		1.00		1.00		1.00	
	IM	1.22	1.13-1.32	1.43	1.20-1.72	0.99	0.84-1.16	1.22	1.00-1.50
**Gender**									
	Male			1.00				1.00	
	Female			1.07	0.98-1.16			1.26	0.99-1.59
**Age**									
	25 and older			1.00				1.00	
	18-24			1.13	1.07-1.21			1.20	1.02-1.42
	17 and younger			1.62	1.50-1.74			1.42	1.17-1.72
**Race**									
	White			1.00				1.00	
	Black			1.40	1.30-1.50			1.12	0.92-1.35
	Latino/Hispanic			1.36	1.27-1.46			1.31	1.10-1.55
	Other			1.34	1.25-1.44			1.35	1.13-1.61
**Conversation topic**									
	Abortion			1.00				1.00	
	STI testing			0.35	0.32-0.39			0.61	0.47-0.78
	Pregnancy testing			0.74	0.69-0.80			0.98	0.82-1.17
	Emergency contraception			0.60	0.53-0.61			1.35	1.13-1.61
	Other			0.40	0.36-0.43			0.97	0.76-1.23
**Planned Parenthood contact info given**									
	No							1.00	
	Yes							0.99	0.87-1.13
**Helpfulness**									
	Less than very helpful							1.00	
	Very helpful							3.74	3.24-4.32

^a^Coefficients represent the odds of feeling very worried prechat as compared to somewhat/not at all worried.

^b^Coefficients represent the odds of feeling less worried after using the service as compared to reporting the same or increased worry.

## Discussion

### Principal Findings

The analysis of the first year of data from the IM and texting program revealed that the program was successful in reaching its target audience, with large portions of users being young (≤24 years), black, and Latino/Hispanic. A large percentage of the study population reported feeling very worried when they initiated the conversation. Because this measure was used as a proxy for whether a user was in a moment of crisis, it is reasonable to assume that the program was also successful in reaching individuals during this vulnerable time. In addition, racial minorities (including black, Latino/Hispanic, and all other groups categorized as other) and users 24 years and younger were more likely to feel very worried when they accessed the program. This may suggest that these groups are in greater need of these types of education and information services. Some evidence from the literature supports this. The 2006-2008 results of the National Survey of Family Growth reported that only 47% of females and 38% of males aged 15-19 years had received information about contraception in high school [8], and research has shown lower levels of knowledge about emergency contraception among individuals aged 18 years and younger [28,29]. Several studies have also documented racial disparities in knowledge levels of sexual and reproductive health issues among US teens and young adults [6,30-32].

Results from the bivariate analysis indicate that the SMS texting service was more likely to be used by younger users and racial minorities than the IM service. This result may simply reflect technology preferences or access of individuals in these groups. This highlights the importance of offering the program through different modes of technology. Although cell phones provide a greater degree of anonymity in the moment, a text conversation, unless deleted, can be reviewed by others who have access to the phone. On the other hand, evidence of a conversation held through IM is gone from the computer once the window is closed, but the computer itself may be in a less private location and the history of visiting the Planned Parenthood website may be accessible to future users of the computer. Because there are continuing advances in technologies with mobile chat, tablets, etc, it is important to consider the implications of these new technologies and their possible impact on program utilization.

The differences that were found between texting and IM users may also be a by-product of the ways that the texting and IM portions of the program were marketed and promoted. The IM portion was only promoted on existing Planned Parenthood Web properties; therefore, it generally reached individuals who were already seeking information online. Recent research has suggested that there are demographic differences in the types of people who seek out health information online [33]. It may be that teens or racial minorities are less likely to search for answers to their sexual and reproductive health questions online, which would cause them to be less likely to be aware of the IM service. Further, the texting service was promoted on the MTV shows *16 and Pregnant* and *Teen Mom*, which appeal to a younger audience. In addition to demographic differences, texting users seemed to be less worried overall than IM users, both before and after the chat session. Again, this may be because of promotion efforts. Because IM users were actively seeking information online, they might already be at a moment of more pressing need. Conversely, individuals who became aware of the texting service while watching MTV may not be as worried when they initially contacted the program. As a result, Planned Parenthood staff may need to reconsider the ways in which the IM portion of the program is marketed and promoted.

Although texting users did appear to be less worried overall, after controlling for the study covariates, there was no difference in the program’s effectiveness in reducing levels of worry postchat for texting versus IM users. This is an encouraging result because it indicates that both conversation modes are equally effective in reducing worry immediately after an individual has interacted with the program. However, because there were differences in the demographic profiles of the IM and texting users, it seems that both modes are needed to effectively reach the target population.

The results of this evaluation indicate that the program was more effective in reducing worry levels for the youngest age group (≤17 years) and for Latino/Hispanics. This result echoes the previous point that these groups may be more in need of this service because of inadequate access to sexual and reproductive health information at home or in school.

Whether or not a user felt better (ie, reduced level of worry) differed greatly by the topic area of their question. As compared to individuals with questions about abortion, the program was more effective in reducing worry when users had questions concerning emergency contraception. On the other hand, the odds of feeling better (ie, reducing worry) postchat were lower for individuals asking about STI testing than for abortion. There are several potential explanations for these differences. It could be that the increased odds of feeling better for individuals with questions about emergency contraception is an indication of a poor understanding of it and its uses in the general population. As such, these individuals may have benefited more from the information they received from the program and were subsequently more relieved. The fact that users with questions about STIs were less likely to report a lower level of worry postchat may be a result of the nature of STI questions. Agents are prohibited from making diagnoses during the conversations, but many individuals present with a list of symptoms and want to know what STI they may have. In these cases, agents are limited to providing information for a health center and advising the user that they will have to wait for several days or weeks after they are tested for results. As a result, users with STI-related questions are likely most frustrated with the lack of information regarding a specific diagnosis.

In an effort to test this theory after initial analysis, Pearson chi-square tests were run to determine if there were significant differences in reporting that the chat was very helpful by question topic area (Table 3), which revealed significant differences in user-reported program helpfulness by question topic. As stated previously, among users with questions concerning STIs, just under half reported that the program was very helpful, representing the smallest percentage of all the question categories. This low percentage could support the argument that users with questions about STIs may be more frustrated with the lack of immediate action or new information that resulted from their conversation, thereby making this group less likely to feel less worried postchat. By comparison, approximately 70% of users with questions about emergency contraception reported that the chat was very helpful, representing the largest percentage of all the question categories. Again, this may suggest users with questions about emergency contraception generally have lower levels of knowledge about this topic area than others and, therefore, found the service to be more helpful.

### Limitations

This study has several limitations. The IM services attracted far more users than the SMS texting service (IM = 27,939 vs texting = 4650). As discussed previously, this is likely partially because of differences in the way that the text and IM services were advertised. This makes it difficult to assess whether the variations in the characteristics of program users by IM or texting are an artifact of these marketing methods or an indication of underlying preferences of users. Further research is needed to more accurately address this issue. In addition, the overall sample is heavily biased toward IM users.

Technological issues also made it difficult to collect data for this evaluation. Program designers did not want to discourage participating or completing the prechat or postchat survey by presenting lengthy surveys. Therefore, gathered information was limited to basic demographics, program helpfulness, and levels of worry prechat and postchat. The worry variables were meant to capture whether or not an individual was in a moment of crisis. However, this is an imperfect proxy because some individuals could have been very worried about their sexual and reproductive health issues for several weeks. Further, as shown in Table 1, technology difference caused data to not be collected in the same way for IM and texting users. Although the race variable was assessed through the prechat survey for IM users, which they were required to fill out; it was only assessed through the postchat survey for texting users. As a result, missing data for the race variable was much higher for texting than IM users. If this missing data was not random, it may have introduced some bias into the study.

### Conclusions

Research has shown that response rates for Internet and text-based surveys are notoriously low [34-36], and the results from this study echo this trend. Response rates for individual question items varied widely from question to question and between the IM and text survey delivery models. In addition, technological limitations of the software program prevented the survey from being offered to all users or from requiring completion by those who were offered it. This problem was most pervasive for the postchat survey. As a result, conclusions about the helpfulness of the program and its effectiveness in reducing worry should be made cautiously. This is especially problematic because one of the main outcome variables for this process evaluation relied on data from this survey. If the individuals who responded to the postchat survey are systematically different from the study sample as a whole, the conclusions drawn from the logistic regression models that investigated changes in postchat worry could be strongly biased. It may be that users who did not find the program helpful or were still very worried about their sexual and reproductive health issue were less likely to respond to the postchat survey.

One of the major goals of the program is to increase access to health services. Program designers hypothesize that providing IM and SMS texting services will help build trust between program users and Planned Parenthood, further encouraging users to seek health services at a Planned Parenthood health center when they are needed. However, funding and technology restrictions did not allow for a more robust follow-up process. Therefore, another limitation of this process evaluation is that it only measures user attitudes immediately after interacting with the program and not behavior change. Further research is needed to systematically measure the impact of the program. This type of evaluation could determine whether individuals who used the program were more likely to utilize health care services or enact positive behavior change than those who did not.

The use of Internet and mobile technology is increasingly becoming an integral part of our everyday interactions and activities. This is especially true among teenagers and young adults. If interventions can be developed that reach young people with information and education that helps reduce worry, encourages the use of needed health services, and motivates changes in health behaviors, these technologies could be an important addition to public health practice.

The results from the process evaluation of the first year of Planned Parenthood Federation of America’s IM- and texting-based intervention offer insight into one possibility for the use of Internet and mobile technologies for sexuality education programs. Although the results are unable to describe the program’s effectiveness in affecting behavior change, they do suggest that the program was able to provide informational support to traditionally vulnerable groups, such as teens and racial minorities, in moments of particular worry.

The differences found between the IM and texting users reveal that each mode appeals to a different population of users and that both are necessary to reach a larger audience. Future research is needed to rigorously evaluate the impact of the program, including whether it increased knowledge of sexual and reproductive health issues, increased the use of health care services, and promoted safer sexual behaviors among users.

## Abbreviations

AOR: adjusted odds ratio
FAQ: frequently asked question
IM: instant messaging
SMS: short message service
STI: sexually transmitted infections
UOR: unadjusted odds ratio
